# The Spectrum of C4d Deposition in Renal Biopsies of Lupus Nephritis Patients

**DOI:** 10.3389/fimmu.2021.654652

**Published:** 2021-07-01

**Authors:** Ying Ding, Xiaojuan Yu, Lihua Wu, Ying Tan, Zhen Qu, Feng Yu

**Affiliations:** ^1^ Renal Division, Department of Medicine, Peking University First Hospital; Institute of Nephrology, Peking University; Key Laboratory of Renal Disease, Ministry of Health of China; Key Laboratory of CKD Prevention and Treatment, Ministry of Education of China, Beijing, China; ^2^ Department of Nephrology, Peking University International Hospital, Beijing, China; ^3^ Department of Nephrology, General Hospital of Ningxia Medical University, Yinchuan, China

**Keywords:** C4d, lupus nephritis, renal microvascular lesions, complement, prognosis

## Abstract

**Objectives:**

This study aimed to determine the prevalence and localization of complement factor C4d in renal biopsies from patients with lupus nephritis (LN), as well as its associations with the disease’s clinico-pathological features. The correlation between arteriolar C4d deposition and renal microvascular lesions (RVLs) was further analyzed.

**Methods:**

A total of 325 biopsy-proven LN patients were enrolled, and their clinico-pathological data were collected. C4d staining of renal biopsies was performed by immunohistochemistry. The associations between C4d deposition and the clinico-pathological features were further analyzed.

**Results:**

C4d deposition was present in most (98.8%) renal specimens in our cohort. These deposits were localized in the glomeruli (98.2%), tubular basement membrane (TBM) (43.7%), arterioles (31.4%), and peritubular capillary (33.8%). Patients with TBM C4d staining had higher disease activity (measured with the Systemic Lupus Erythematous Disease Activity Index) and higher National Institutes of Health pathological activity and chronicity indices (all *P* < 0.01). Patients with arteriolar C4d deposition were more likely to develop RVLs (91.2%) compared to those with no arteriolar C4d deposition (78.0%; *P* = 0.004), especially with two or more types of RVLs (*P* < 0.001). During the mean follow-up of 55.8 months, arteriolar C4d was related to worse renal outcomes [hazard ration (HR): 2.074, 95% confidence interval (CI) 1.056–4.075, *P* = 0.034]. Multivariate Cox hazard analysis showed that co-deposition of arteriolar C4d and C3c was an independent risk factor (HR: 3.681, 95% CI 1.519–8.921, *P* = 0.004) for predicting renal outcomes.

**Conclusions:**

C4d deposition was common in renal tissues from LN patients. TBM C4d deposition was related to the disease activity, and arteriolar C4d deposition was associated with RVLs and worse renal outcomes.

## Introduction

Systemic lupus erythematous (SLE) is an autoimmune disease with a wide variety of clinical manifestations and serological abnormalities (1). Lupus nephritis (LN) presents in up to 60% of patients during the disease course (2) and contributes to SLE’s morbidity and mortality. Renal histopathology is closely related with its clinical characteristics, responses to treatment, and patient prognosis (3).

Complement activation plays a key role in LN pathogenesis (4). The classical pathway is thought to be the dominant pathway for complement activation in LN, which is triggered by the interaction of C1q with immune complexes (5). Several studies detected mannose-binding lectin (MBL) in the glomeruli of LN patients (6, 7), implicating its participation in disease progression.

C4d is a fragment of C4 generated during activation of the complement pathway and is regarded as a hallmark of classical or lectin complement pathway activation (8). Its deposition in renal peritubular capillaries (PTCs) is widely accepted as a marker of antibody-mediated rejection (AMR) in transplanted kidneys (9, 10). Its meaning in other kidney diseases like LN in which many different autoantibodies produced an analogous situation has also been investigated in recent years. C4d deposition on PTCs has been observed in LN patients, but with a different pattern from those observed in antibody-mediated renal rejection, and this event is closely related to the disease activity (11–13). Besides, Kim et al. found that glomerular C4d deposition could be detected in the majority of the LN cases, but concluded that it was not a marker for LN activity (14). However, another study conducted by Cohen and colleagues demonstrated that LN patients with prominent diffuse glomerular C4d deposition were more likely to develop renal thrombotic microangiopathy (TMA) lesions than patient with focal or mild C4d staining (15). Thus, the role of C4d deposition, especially in the different areas of renal biopsies in LN, remains to be further clarified.

Herein, we assessed the prevalence and anatomic localization of C4d deposition in renal biopsy specimens from a well-defined LN cohort. The associations between C4d deposition in different kidney compartments and clinico-histopathological features and outcomes of LN were further analyzed.

## Methods

### Patients

Clinical and renal histopathological data of 325 patients with renal biopsy proven LN diagnosed between January 2000 and June 2008 from Peking University First Hospital were collected. All the patients included fulfilled the 1997 American College of Rheumatology revised criteria for SLE (16). Patients who lacked medical record information or follow-up data (n = 37), lacked renal biopsy samples for re-examination (n = 20) or had <10 glomeruli and <6 vessels in renal biopsies (n = 9) were excluded. More details on the study design are presented as a flowchart in **Supplementary Figure 1**. The biopsies and biologic samples used in this study came from the biorepository of the Department of Nephrology, Pekin University First Hospital. Informed consent was obtained from each patient for blood sampling and renal biopsy. The research was in compliance with the Declaration of Helsinki. The protocol was approved by the local ethical committees of Peking University First Hospital [No. 2017 (1333)].

### Clinical Evaluation

Disease activity was assessed with the Systemic Lupus Erythematous Disease Activity Index (SLEDAI) (17, 18). All patients were followed up in outpatient clinics specified for LN. Acute kidney injury is defined as an increase in serum creatinine (Scr) by 50% within 7 days, an increase in Scr by 0.3 mg/dl (26.5 μmol/L) within 2 days, or oliguria (19). The end point was defined as end stage renal disease (ESRD) or doubling of the Scr value. ESRD was defined as requiring renal replacement therapy (20).

### Laboratory Assessment

Serum antinuclear antibodies were detected using the indirect immunofluorescence assay (EUROIMMUN, Lübeck, Germany). Anti-double-stranded DNA (anti-dsDNA) antibodies were detected using the Crithidia luciliae indirect immunofluorescence test (EUROIMMUN). Serum C3 and C4 were determined using the rate nephelometry assay (IMMAGE; Beckman-Coulter, IMMAGE, Fullerton, CA; normal range >0.85 g/L for C3, normal range >0.12 g/L for C4). Anti-cardiolipin antibodies (aCL) were detected using enzyme-linked immunosorbent assay (EUROIM MUN).

### Renal Histopathology

The renal biopsy specimens were routinely examined by light microscopy, direct immunofluorescence, and electron microscopy techniques. Renal histopathology data were reviewed and reclassified according to the International Society of Nephrology and Renal Pathology Society 2003 LN classification system (21) by two experienced pathologists. They independently reviewed the biopsy specimens separately and were blinded to patients’ clinical and follow-up data. If there were differences in scoring between the two pathologists, they would re-review the biopsies and reach a consensus. Pathological parameters such as activity indices (AIs) and chronicity indices (CIs) were evaluated as described previously (22). Activity indices was defined as the sum of individual scores of the following items: endocapillary hypercellularity, cellular crescents (×2), karyorrhexis/fibrinoid necrosis (×2), subendothelial hyaline deposits, interstitial inflammatory cell infiltration, and glomerular leukocyte infiltration. The maximum score was 24 points for the Activity Index. Chronicity indices was defined as the sum of individual scores of the following items: glomerular sclerosis, fibrous crescents, tubular atrophy, and interstitial fibrosis. The maximum score was 12 points for the Chronicity Index (22). Renal microvascular lesions (RVLs) were classified according to the previous studies (23, 24) as follows: immune complex deposits (ICD), non-inflammatory necrotizing vasculopathy (NNV), thrombotic microangiopathy (TMA), true renal vasculitis (TRV), and atherosclerosis (AS). Renal TMA lesions were subdivided into active changes and chronic changes. Active TMA lesion was defined as the presence of at least one fibrin microthrombus either in the glomeruli, small arterioles, and/or arteries. Chronic TMA changes were defined as mucoid changes or onion-skin lesions of arterioles and/or arteries (24). Semiquantitative RVL scores were calculated as described in our previous work (24).

### C4d, C1q, C3c, and Immunoglobulin Staining

**Figure 1:**
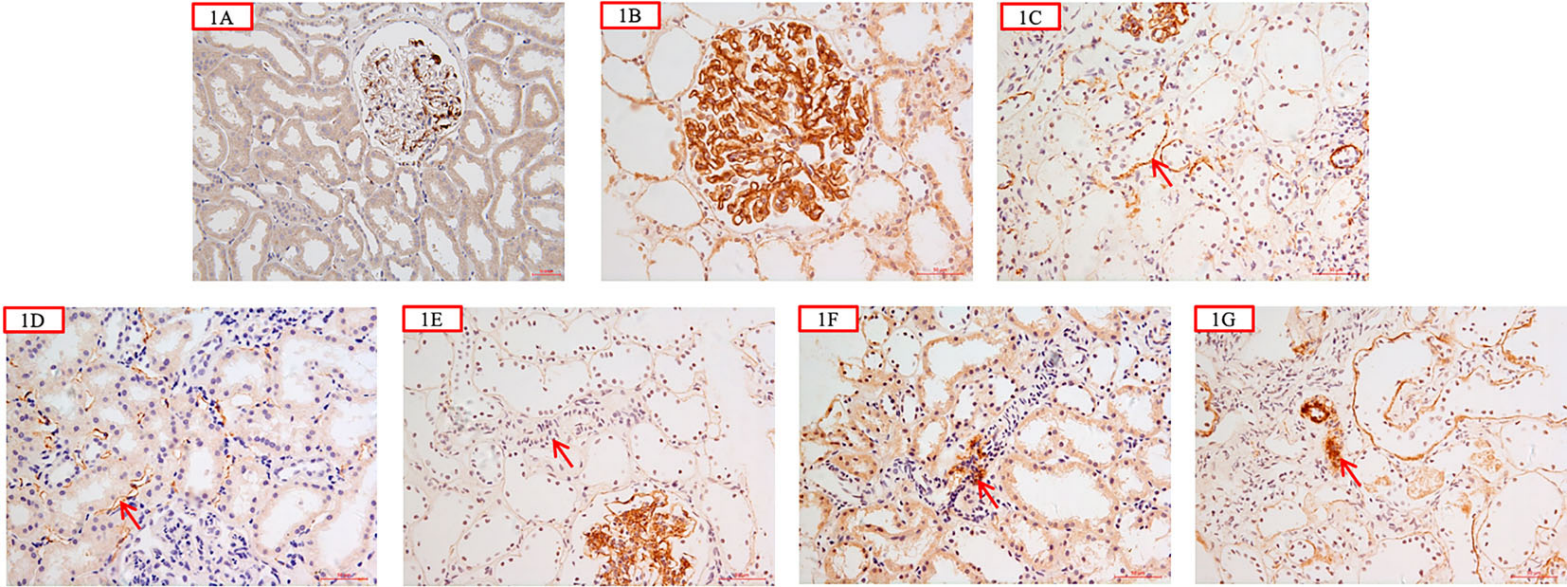
**(A)** was negative control from a living kidney donor which showed scanty granular mesangial C4d deposition; C4d was negative along tubular basement membrane (TBM) and peritubular capillary wall (PTC) (×200); **(B)** showed glomerular C4d granular deposition along the capillary wall and in the mesangial area (×400); **(C)** showed C4d deposition along the tubular basement membrane (red arrow) (×400); **(D)** showed C4d deposition along peritubular capillary wall (red arrow) (×400); **(E)** showed that C4d was negative in the arteriolar wall (red arrow) (×400); **(F)** showed C4d segmental arteriolar wall deposition (red arrow) (×400); **(G)** showed C4d deposition in the arteriolar wall (red arrow) (×400).

C4d was immunohistochemically labeled in formalin-fixed paraffin-embedded tissue (4-μm thick). Rabbit anti-human C4d polyclonal antibodies (Abcam, Cambridge, UK) were used as primary antibodies. The intensity of Glomerular C4d (G-C4d) was semi-quantitatively graded (0–3) (12, 25). Tubular basement membrane (TBM) C4d (TBM-C4d) was considered to be present if more than half of the individual TBM circumference was stained. It was then graded as negative (0%), minimal (1, <10% of tissue specimen), focal (2, 10–50% of tissue specimen), or diffuse (3, >50% of tissue specimen) (12, 25). PTC-C4d staining was graded in accordance with the Banff 2007 criteria for C4d staining as negative (0%), minimal (1, <10% of tissue specimen), focal (2, 10–50% of tissue specimen), or diffuse (3, >50% of tissue specimen) (26). Arteriolar C4d staining (A-C4d) was semi-quantitatively graded as negative (0%), minimal (1, <10% of tissue specimen), focal (2, 10–50% of tissue specimen), or diffuse (3, >50% of tissue specimen) (12, 27). Negative control experiments were performed on the renal specimens of a living kidney donor. Blank control experiments were performed by omitting or replacing the primary antibodies. The typical presentations of C4d staining in different areas of kidneys are shown in **Figure 1**.

The fresh frozen tissue sections were stained immediately after the renal biopsy with fluorescein isothiocyanate-labeled rabbit anti-human IgG, IgA, IgM, C3c, and C1q antibodies (Dako A/S, Copenhagen, Denmark). The results were graded from 0 to 4 according to the intensity of fluorescence.

### Statistical Analysis

Statistical software SPSS 18.0 (SPSS, Chicago, IL, USA) and GraphPad-Prism 5.0 (GraphPad Software, La Jolla, CA, USA) were used for statistical analyses. Quantitative data are expressed as mean ± s.d. or median with range (minimum and maximum) or interquartile range. Spearman’s rank-order correlation tests were performed to compare various lesions. Differences in semiquantitative data were tested with the Kruskal–Wallis test or Mann–Whitney U-tests. Bonferroni’s correction was used for multiple comparisons. Kaplan–Meier curves were generated to analyze patients’ prognosis. Univariate survival analysis was carried out using the log-rank tests. The Cox regression model was applied to identify prognostic factors associated with renal outcomes. The proportional hazards assumption was tested by using Schoenfeld residuals. No violations were found for any of the covariates based on a *P* value threshold of 0.05. Results are expressed as hazard ratios (HR) with 95% confidence intervals (CI). Statistical significance was considered at P < 0.05.

## Results

### Baseline Data of the LN Cohort

A total of 325 LN patients were enrolled. Their clinico-pathological data are presented in **Table 1**.

**Table 1:** Baseline clinico-pathological data of lupus nephritis patients.

Number of patients	325
**Gender (Female/Male)**	271/54
**Age (mean ± s.d.) (years)**	32.8 ± 11.5
**Hypertension, n(%) (blood pressure≧140/90mmHg)**	166 (51.1%)
**Pleuritis, n(%)**	55 (16.3%)
**Neurological disorder, n(%)**	25 (7.7%)
**Anemia, n(%)**	219 (67.4%)
**Thrombocytopenia, n(%)**	104 (32.0%)
**Hematuria, n(%)**	249 (76.6%)
**Acute kidney injury, n(%)**	67 (20.6%)
**Urine protein (median and range) (g/24h)**	4.3 (0-21.0)
**Serum creatinine value (median and range) (μmmol/l)**	83.0 (37.1-971.0)
**C3 (median, IQR) (g/L)**	0.43 (0.32-0.62)
C4 (median, IQR) (g/L)^*^	0.11 (0.04-0.16)
**ANA positivity, n(%)**	320 (98.5%)
**Anti-dsDNA Ab positivity, n(%)**	213 (65.5%)
**Numbers with positive aCL, n(%)^#^**	19/198 (9.6%)
**SLEDAI (mean ± s.d.)**	17.4 ± 5.8
**Class II (%)**	19 (5.8%)
**Class III (%)**	67 (20.6%) (28 III+V)
**Class IV (%)**	178 (54.8%) (33 IV+V)
**Class V (%)**	59 (18.2%)
**Class VI (%)**	2 (0.6%)
**AI score (median; IQR)**	8; 4-11
**Endocapillary hypercellularity (median; IQR)**	3; 1-3
**Cellular crescents (median; IQR)**	0; 0-2
**Karyorrhexis/fibrinoid necrosis (median; IQR)**	0; 0-2
**Subendothelial hyaline deposits (median; IQR)**	1; 0-2
**Interstitial inflammatory cell infiltration (median; IQR)**	1; 1-2
**Glomerular leukocyte infiltration (median; IQR)**	1; 0-1
**CI score (median; IQR)**	2; 2-4
**Glomerular sclerosis (median; IQR)**	0; 0-1
**Fibrous crescents (median; IQR)**	0; 0-0
**Tubular atrophy (median; IQR)**	1; 1-1
**Interstitial fibrosis (median; IQR)**	1; 1-1
**Renal microvascular lesions**	267 (82.2%)
**ICD (%)**	240 (73.8%)
**AS (%)**	84 (25.8%)
**TMA (%)**	58 (17.8%)
**NNV (%)**	13 (4.0%)
**TRV (%)**	2 (0.6%)
**Treatment**	
**Oral prednisone**	325 (100%)
**Cyclophosphamide**	254 (78.2%)
**Mycophenolate mofetil**	27 (8.3%)
**Leflunomide**	12 (3.7%)
**Azathioprine**	3 (0.9%)
**Cyclosporine**	1 (0.3%)

ANA, antinuclear antibody; Anti-dsDNA Ab, Anti-double-stranded DNA antibody; aCL, anti-cardiolipin antibody; SLEDAI, systemic lupus erythematosus disease activity index; AI, activity index; CI, chronicity index; ICD, immune complex deposits; AS, atherosclerosis; TMA, thrombotic microangiopathy; NNV, noninflammatory necrotizing vasculopathy; TRV, true renal vasculitis; n, number; s.d., standard deviation; IQR, interquartile range.*C4 was analyzed based on the data of 239 patients with C4 level tested at the onset of the disease; ^#^aCL was analyzed based on the data of 198 patients with aCL level tested at the onset of the disease.

All 325 patients received oral prednisone therapy. Among them, 28 patients received prednisone alone, 254 patients received cyclophosphamide [238 patients received the monthly intravenous cyclophosphamide (600–800 mg/months), and 16 patients received oral cyclophosphamide], 27 patients received mycophenolate mofetil, 12 patients received leflunomid, 3 patients received azathioprine, and 1 patient received cyclosporine.

The average follow-up time was 55.8 months (range from 4 to 360 months). During this period, three patients died of heart failure and one patient died of severe infection. A total of 37 patients reached the end point, including 35 with ESRD and 2 with Scr doubling.

### The Prevalence and Localization of C4d Staining in Renal Specimens of LN Patients

C4d staining was present in 321/325 (98.8%) LN samples (**Table 2**). They were localized in the glomerulus (319/325, 98.2%), TBM (142/325, 43.7%), arterioles (102/325, 31.4%), and PTCs (110/325, 33.8%) respectively.

**Table 2:** C4d staining patterns in different renal anatomic compartments.

	Glomeruli	Arteriole	PTC	TBM
**Negative**	6 (1.8%)	223 (68.6%)	215 (66.2%)	183 (56.3%)
**Minimal**	37 (11.4%)	78 (24%)	90 (27.7%)	116 (35.7%)
**Focal**	88 (27.1%)	15 (4.6%)	13 (4.0%)	21 (6.5%)
**Diffuse**	194 (59.7%)	9 (2.8%)	7 (2.2%)	5 (1.5%)

PTC, peritubular capillary; TBM, tubular basement membrane; Minimal, Focal and Diffuse represent the distribution of the C4d staining <10%, 10-50% and >50% respectively for Arteriole, PTC and TBM. Minimal, Focal and Diffuse represent the semi-quantitative grade (1-3) of the glomerular C4d staining for Glomeruli.

**Figure 2:**
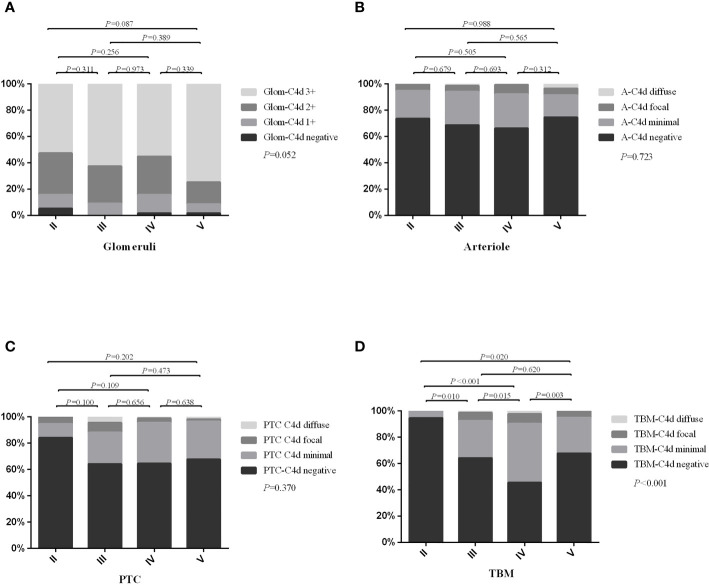
The distribution or intensity of C4d staining between different lupus nephritis sub-classes. **(A)** showed the intensity of glomerular C4d staining; **(B)** showed distribution of arteriolar C4d staining; **(C)** showed distribution of PTC-C4d staining; **(D)** showed distribution of TBM-C4d staining. Glom, glomerular; A, arteriolar; PTC, peritubular capillary; TBM, tubular basement membrane. Bonferroni’s correction for multiple correlations: statistical significance was considered at *P* < 0.008 (0.05/6).

The intensity or distribution of C4d staining among different sub-classes of LN are shown in **Figure 2**. We found that the degree of TBM C4d deposition significantly differed among the subtypes (*P* < 0.001); it was more often present in patients with classes III, IV, and V disease than class II. The rates of glomerular, arteriolar, and PTC C4d deposition were not significantly different among the subgroups.

### Comparison of C4d Deposition in Different Renal Compartments With Clinico-Pathological Data

The detailed analyses between C4d deposition in different renal areas and clinical and laboratory features are shown in **Table 3**. Notably, hematuria (*P* = 0.035), leukocyturia (*P* = 0.002), and acute kidney injury (*P* = 0.001) were more frequent in TBM C4d-positive patients, who also had higher levels of urinary protein, Scr, and SLEDAI value, and a lower serum C3 value.

**Table 3:** Comparison of clinical and laboratory data in patients with and without C4d deposition in different renal compartments.

	Glomeruli			Arteriole			PTC			TBM		
	0/1+43pt	2+/3+282pt	*P*	Negative 223pt	Positive 102pt	*P*	Negative 215pt	Positive 110pt	*P*	Negative 183pt	Positive 142pt	*P*
**Gender (male/female)**	8/35	46/236	0.707	40/183	14/88	0.423	40/175	14/96	0.209	34/149	20/122	0.297
**Age (year) (mean±s.d.)**	33.81±12.62	32.59±11.38	0.665	32.43±11.42	33.46±11.83	0.456	31.90±11.47	34.43±11.55	0.061	32.38±11.10	33.23±12.10	0.511
**Number with hematuria (%)**	36 (83.7%)	213 (75.5%)	0.238	171 (76.7%)	78 (76.5%)	1.000	160 (74.4%)	89 (80.9%)	0.214	132 (72.1%)	117 (82.4%)	0.035
**Numbers with leukocyturia (non-infectious) (%)**	23 (53.5%)	150 (53.2%)	0.990	117 (52.5%)	56 (54.9%)	0.721	115 (53.5%)	58 (52.7%)	0.907	83 (45.4%)	90 (63.4%)	0.002
**Urine protein (g/24h) (median, IQR)**	3.34 (1.61-4.88)	4.45 (2.38-7.00)	0.031	4.30 (2.04-6.82)	4.34 (2.75-6.90)	0.238	4.26 (2.04-6.39)	4.46 (2.77-8.00)	0.084	4.00 (1.87-6.30)	4.84 (2.86-7.62)	0.004
**Numbers with acute kidney injury (%)**	9 (20.9%)	56 (19.9%)	0.870	41 (18.4%)	24 (23.5%)	0.298	41 (19.1%)	24 (21.8%)	0.561	25 (13.7%)	40 (28.2%)	0.001
**Serum creatinine (μmmol/l) (median, IQR)**	84 (68-157)	82 (68-131)	0.550	79 (66-115)	92 (73-189)	0.002	80 (66-119)	87 (72-169)	0.014	78 (65-98)	99 (73-187)	<0.001
**C3 (g/L) (median, IQR)**	0.35 (0.19-0.48)	0.44 (0.34-0.63)	<0.001	0.42 (0.29-0.62)	0.44 (0.33-0.63)	0.528	0.45 (0.32-0.65)	0.41 (0.30-0.51)	0.036	0.46 (0.33-0.65)	0.41 (0.28-0.56)	0.014
**C4 (g/L) (median, IQR)^#^**	33 patients 0.04 (0.025-0.125)	206 patients 0.11 (0.05-0.17)	0.001	176 patients 0.095 (0.033-0.16)	63 patients 0.12 (0.08-0.17)	0.027	162 patients 0.11 (0.04-0.17)	77 patients 0.10 (0.05-0.15)	0.442	135 patients 0.10 (0.03-0.17)	104 patients 0.11 (0.06-0.15)	0.481
**Numbers with ANA Positivity (%)**	43 (100%)	277 (98.2%)	0.380	220 (98.7%)	100 (98.0%)	0.651	211 (98.1%)	109 (99.1%)	0.666	179 (97.8%)	141 (99.3%)	0.391
**Numbers with positive Anti-dsDNA Ab (%)**	35 (81.4%)	178 (63.1%)	0.022	149 (66.8%)	64 (62.7%)	0.528	136 (63.3%)	77 (70.0%)	0.217	115 (62.8%)	98 (69.0%)	0.240
**Numbers with positive aCL (%)^##^**	2/24 (8.3%)	17/174 (9.8%)	0.823	17/139 (12.2%)	2/59 (3.3%)	0.054	11/131 (8.4%)	8/67 (11.9%)	0.424	11/109 (10.1%)	8/89 (9.0%)	0.794
**SLEDAI (median, IQR)**	18 (15-23)	17 (13-21)	0.172	17 (13-21)	17 (13-21)	0.882	17 (13-20)	18 (14-22)	0.096	16 (12-20)	18 (15-22)	0.001

Glomeruli was divided into 0/1+ and 2+/3+ subgroups according to the glomerular C4d intensity; Arteriolar, PTC and TBM was divided into negative and positive subgroups according to the C4d positivity. Pt, patients; PTC, peritubular capillary; TBM, tubular basement membrane; ANA, antinuclear antibody; Anti-dsDNA Ab, Anti-double-stranded DNA antibody; aCL, anti-cardiolipin antibody; SLEDAI, systemic lupus erythematosus disease activity index. ^#^C4 was analyzed based on the data of 239 patients with C4 level tested at the onset of the disease; ^##^aCL was analyzed based on the data of 198 patients with aCL level tested at the onset of the disease.

Renal histopathological findings showed that C4d deposition in TBM, arterioles, and PTCs area had higher NIH activity indices (AI) and chronic indices (CI) than those without C4d deposition in the corresponding areas. Detailed data were presented in **Table 4**.

**Table 4:** Comparison of renal histopathological data in patients with and without C4d deposition in different renal compartments.

	Glomeruli			Arteriole			PTC		TBM			
	0/1+ 43pt	2+/3+ 282pt	*P*	Negative 223pt	Positive 102pt	*P*	Negative 215pt	Positive 110pt	*P*	Negative 183pt	Positive 142pt	*P*
**AI score**	9 (5-13)	7 (3-11)	0.102	7 (3-11)	8 (5-12)	0.023	7 (3-11)	9 (5-12)	0.011	5 (2-10)	9 (6-12)	<0.001
**Endocapillary hypercellularity**	3 (2-3)	2 (1-3)	0.243	2 (1-3)	3 (2-3)	0.067	2 (1-3)	3 (1-3)	0.184	2 (1-3)	3 (2-3)	<0.001
**Cellular crescents**	0 (0-2)	0 (0-2)	0.238	0 (0-2)	2 (0-2)	0.003	0 (0-2)	2 (0-2)	0.041	0 (0-2)	2 (0-4)	<0.001
**Karyorrhexis/fibrinoid necrosis**	2 (0-2)	0 (0-2)	0.021	0 (0-2)	0.5 (0-2)	0.311	0 (0-2)	2 (0-2)	0.037	0 (0-2)	2 (0-2)	0.014
**Subendothelial hyaline deposits**	1 (0-3)	1 (0-2)	0.078	1 (0-2)	1 (0-2)	0.631	1 (0-2)	1 (1-2)	0.040	1 (0-2)	1 (0-2)	0.037
**Interstitial inflammatory cell infiltration**	1 (1-2)	1 (1-1.25)	0.142	1 (1-1)	1 (1-2)	0.014	1 (1-1)	1 (1-2)	0.003	1 (1-1)	1 (1-2)	<0.001
**Glomerular leukocyte infiltration**	1 (1-1)	1 (0-1)	0.064	1 (0-1)	1 (0-1)	0.374	1 (0-1)	1 (0-1)	0.330	1 (0-1)	1 (0-1)	0.004
**CI score**	2 (2-3)	2 (2-4)	0.563	2 (1-3)	3 (2-4)	0.001	2 (1-3)	3 (2-4)	<0.001	2 (1-3)	3 (2-4)	<0.001
**Glomerular sclerosis**	0 (0-1)	0 (0-1)	0.041	0 (0-1)	0 (0-1)	0.022	0 (0-1)	0 (0-1)	0.019	0 (0-1)	0 (0-1)	0.134
**Fibrous crescents**	0 (0-0)	0 (0-0)	0.778	0 (0-0)	0 (0-0)	0.541	0 (0-0)	0 (0-0)	0.101	0 (0-0)	0 (0-0)	0.007
**Tubular atrophy**	1 (1-1)	1 (1-1)	0.637	1 (1-1)	1 (1-2)	<0.001	1 (1-1)	1 (1-2)	<0.001	1 (1-1)	1 (1-2)	<0.001
**Interstitial fibrosis**	1 (0-1)	1 (1-1)	0.685	1 (0-1)	1 (1-1)	<0.001	1 (0-1)	1 (1-1)	0.003	1 (0-1)	1 (1-1)	<0.001

Pt, patients; PTC, peritubular capillary; TMB, tubular basement membrane; AI, activity indices; CI, chronicity indices. Data were presented as median (interquartile range) in the table.

### Association Between Arteriolar C4d (A-C4d) Deposition and RVLs in LN Patients

**Figure 3:**
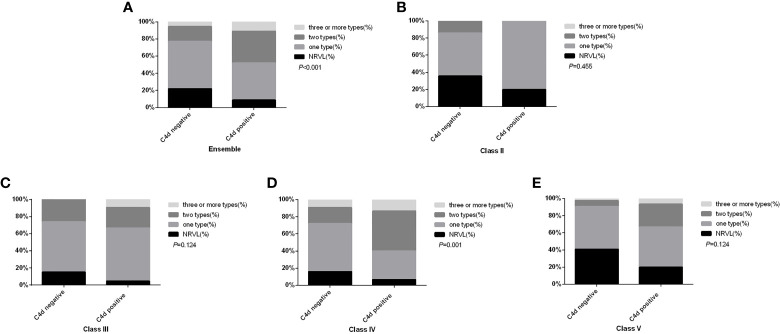
Comparison of renal microvascular lesion types between patients with and without arteriolar C4d deposition in the ensemble **(A)**, in subgroup class II **(B)**, in subgroup class III **(C)**, in subgroup class IV **(D)**, and in subgroup class V **(E)**. NRVL, no renal microvascular lesions.

RVLs were common in LN, so the relationship between arteriolar C4d (A-C4d) deposition and RVLs was explored. In our study, 267 (82.2%) patients presented with RVLs. Among them, 34.8% of patients had A-C4d deposition, which was a much higher rate than in those without RVL (15.5%, *P* = 0.004). Similarly, those with A-C4d deposition had a higher rate of RVLs (91.2%) compared to those negative for A-C4d (78.0%; *P* = 0.004), especially multiple RVLs (*P* < 0.001), which is most obvious in type IV LN (*P* = 0.001) (**Figure 3**). Moreover, diffuse (>50%) or focal (10–50%) renal A-C4d staining patterns were more common in patients with RVLs [n = 22/267 (8.2%)] compared with those without [n = 2/58 (3.4%); *P* = 0.035].

With regard to RVL subtypes, patients with TMA lesions had the highest rate of A-C4d deposition (48.3%), followed by patients with ICD (38.3%), NNV (38.5%), and AS (34.5%). Significantly more C4d deposition was found in patients with TMA lesions (48.3%, 28/58) or ICD lesions (38.3%, 92/240) compared to those without TMA lesions (27.7%, 74/267, *P* = 0.003) or ICD lesions (11.8%, 10/85, *P* < 0.001). No significant differences were found for AS, NNV, and TRV lesions. Furthermore, among 58 patients with TMA lesions, 25 had acute TMA, 14 had chronic TMA, and 19 had both acute and chronic (A+C) TMA lesions. Arteriolar C4d deposition was not found significantly different amongst the groups: 44.0% (11/25) in acute TMA subgroup, 42.1% (8/19) in A+C TMA, and 64.3% (9/14) in chronic TMA cases (*P* = 0.385). Neither C4d deposition nor the presence of renal TMA lesions correlated with aCL status: aCL was positive in 2.8% (1/36) in TMA group and 11.1% of (18/162) patients without TMA (P = 0.208). ACL was positive in 3.4% (2/59) of arteriolar C4d positive patients and in 12.2% (17/139) of arteriolar C4d negative patients (P = 0.065).

Analysis of co-depositions of A-C4d and C1q, C3c and different immunoglobulins were further analyzed. Patients with A-C4d deposits had higher ratios of C1q deposition (75.5% *vs.* 59.2%, *P* = 0.004), C3c deposition (39.2% *vs.* 28.7%, *P* = 0.059), IgG deposition (40.2% *vs.* 29.1%, *P* = 0.049), IgM deposition (37.3% *vs.* 25.6%, *P* = 0.031), and IgA deposition (17.6% *vs.* 7.2%, *P* = 0.004) compared to those without A-C4d deposits. Patients with both C4d and C3c staining had significantly higher scores of CI (*P* = 0.024), tubular atrophy (*P* = 0.049), and interstitial fibrosis (*P* = 0.027) than those who were only C4d positive. Similar results were also found in the C4d+C1q, C4d+IgG, C4d+IgM, and C4d+IgA double positive groups (**Supplementary Table 1**).

### C4d Deposition and Renal Outcomes in LN

**Figure 4:**
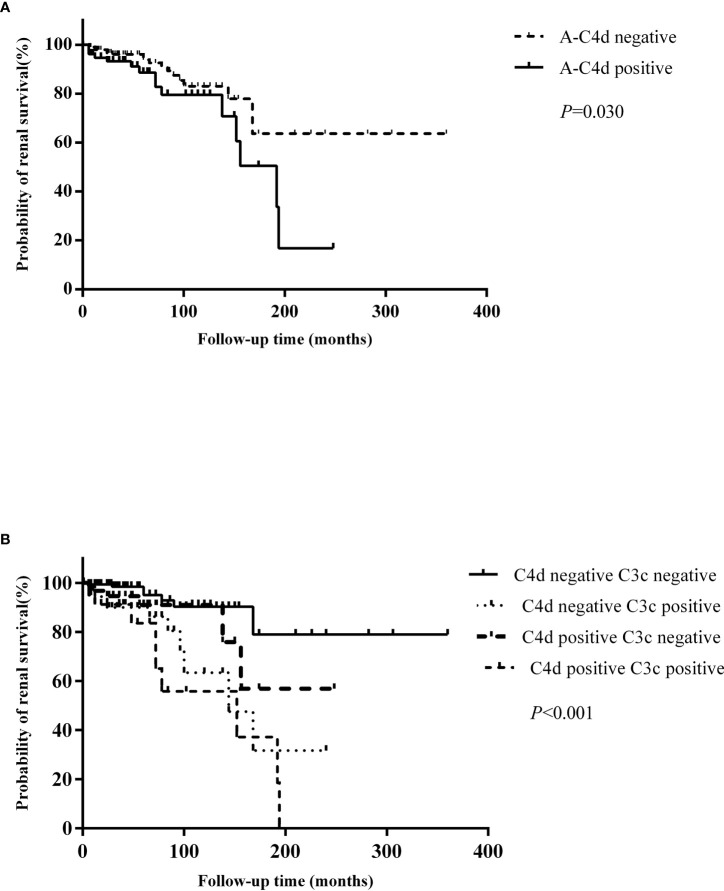
**(A)** Comparison of renal outcomes between patients with and without arteriolar C4d deposition. **(B)** Comparison of renal outcomes between patients with and without arteriolar C4d and C3c deposition.

We found that patients with arteriolar C4d deposition (*P* = 0.03) presented with worse renal survival rates than those who were negative (**Figure 4A**). C4d deposition in other renal compartments did not influence patient renal outcomes. When the patients were categorized into four groups according to the arteriolar C4d and C3c staining, those with both arteriolar C4d and C3c staining had the worst renal outcomes (**Figure 4B**).

Univariate survival analysis of renal prognosis in LN revealed that arteriolar C4d (HR 2.074, 95% CI 1.056–4.075, *P* = 0.034) deposition and co-deposition of C4d and C3c (HR 3.652, 95% CI 1.736–7.683, *P* = 0.001) were risk factors for poor renal outcomes (**Table 5**). Subsequent multivariate Cox hazard analysis showed that C4d deposition and the co-deposition of C4d and C3c were both independent risk factors for predicting renal outcomes in LN (**Table 6**).

**Table 5:** Univariate survival analysis of patients’ renal prognosis in lupus nephritis.

	Hazard ratio	95% Confidence intervals		P-Value
		Lower	Upper	
Sex (Male *vs.* Female)	0.414	0.175	0.981	0.045
Age (per year)	0.977	0.946	1.009	0.155
Hypertension (present *vs.* absent)	0.504	0.240	1.055	0.069
Neurological disorder (present *vs.* absent)	1.439	0.506	4.094	0.495
Thrombocytopenia (present *vs.* absent)	1.012	0.483	2.120	0.974
Serum creatinine (per μmol/L)	1.007	1.005	1.008	<0.001
Acute renal injury (present *vs.* absent)	8.097	4.031	16.263	<0.001
Proteinuria (per g/24 hours)	1.046	0.960	1.140	0.300
Anti-dsDNA antibodies (present *vs.* absent)	2.501	1.108	5.647	0.027
aCL (present *vs.* absent)	0.903	0.253	4.742	1.096
Serum C4 level (per g/L)	0.910	0.157	5.287	0.916
Glomerular C4d intensity (per intensity point)	0.718	0.496	1.037	0.077
TBM C4d deposition (present *vs.* absent)	1.558	0.783	3.101	0.207
Arteriolar C4d deposition (present *vs.* absent)	2.074	1.056	4.075	0.034
Arteriolar C4d and C3c deposition (present *vs.* absent)	3.652	1.736	7.683	0.001
PTC C4d deposition (present *vs.* absent)	1.045	0.522	2.093	0.901
SLEDAI (per point)	1.017	0.961	1.077	0.555
AI score (per point)	1.181	1.090	1.280	<0.001
CI score (per point)	1.371	1.205	1.559	<0.001
Renal microvascular lesions score (per point)	1.419	1.073	1.876	0.014
Treatment				
Prednisone alone (reference)	1.0			
Cyclophosphamide (IV)	1.617	0.386	6.774	0.511
Cyclophosphamide (oral)	0.618	0.056	6.831	0.695
MMF	2.316	0.420	12.780	0.335
**LN sub-class**				
III (reference)	1.0			
IV	12.160	1.659	89.133	0.014
V	2.195	0.199	24.214	0.521

dsDNA, double-stranded DNA; aCL, anti-cardiolipin antibody; SLEDA, Systemic Lupus Erythematosus Disease Activity Index; AI, activity indices; CI, chronicity indices; IV, intravenous; MMF, mycophenolate mofetil; LN, lupus nephritis.

**Table 6:** Multivariate survival analysis of the risk factors for renal outcome in patients with lupus nephritis patients.

	Hazard ratio	95% Confidence intervals		P-Value
		Lower	Upper	
Multivariate Cox hazard analysis				
Sex (Male *vs.* Female)	0.289	0.109	0.767	0.013
Age (per year)	0.989	0.957	1.023	0.538
Acute kidney injury (present *vs.* absent)	3.206	1.127	9.118	0.029
Anti-dsDNA antibodies (present *vs.* absent)	1.999	0.778	5.135	0.150
Arteriolar C4d deposition (present *vs.* absent)	2.260	1.068	4.782	0.033
Arteriolar C3c deposition (present *vs.* absent)	2.089	0.927	4.709	0.076
AI score (per point)	1.040	0.927	1.166	0.506
CI score (per point)	1.250	1.049	1.489	0.013
Multivariate Cox hazard analysis				
Sex (Male *vs.* Female)	0.302	0.114	0.803	0.016
Age (per year)	0.985	0.951	1.019	0.379
Acute kidney injury (present *vs.* absent)	3.142	1.112	8.879	0.031
Anti-dsDNA antibodies (present *vs.* absent)	2.227	0.846	5.862	0.105
Arteriolar C4d and C3c deposition (present *vs.* absent)	3.681	1.519	8.921	0.004
AI score (per point)	1.054	0.940	1.183	0.369
CI score (per point)	1.277	1.083	1.504	0.004

dsDNA, double-stranded DNA; AI, activity indices; CI, chronicity indices.

## Discussion

Complement activation, especially *via* the classical pathway, is widely accepted to play an important role (28) in the LN pathogenesis (29). C4d is a fragment of complement activation that remains covalently bound long after the complement pathway-initiating factors have dissociated. It can be generated by immune complexes through both the classical and lectin pathways (30, 31) and might play a key role in the pathomechanisms of the disease (32). However, there is a lack of generic descriptions of its distribution in renal biopsies from patients with LN; studies of larger cohorts and more detailed analyses of its clinico-pathological significance are needed.

In this study, renal C4d deposition was ubiquitous (98.8%) in LN patients, which was mildly higher than the previous reported values ranging from 86.8 to 92% (12, 15, 33). The difference in C4d deposition rates between studies might be attributable to the varied testing methods and the disease activity of the patients enrolled (12, 25); more than half of our patients had type IV LN with a rather high disease activity. Interestingly, the C4d deposition pattern and rate varied among different renal compartments, reflecting clinico-pathological features. TBM C4d deposition was most strongly related to disease activity, while arteriolar C4d deposition had prognostic value for the renal outcomes.

Glomerular C4d deposition was common in LN in both our study and previous reports (12, 15, 28). Sahin and colleagues suggested that glomerular C4d staining could be an indicator of disease activity in LN patients (34). Cohen et al. and Shen et al. also reported that the intense glomerular C4d staining was an indicator of thrombotic microangiopathy in LN (15, 28). However, neither its distribution nor intensity was related to disease activity or any RVLs in our study, which is supported by the work by Kim et al. and Batal et al., indicating that its deposition may simply reflect *in situ* classical complement pathway activation induced by immune complexes (12, 14).

PTC-C4d deposition is a key marker for diagnosing antibody-mediated renal allograft rejection, and it can also be detected in native kidneys (35). Interestingly, a study from China found PTC-C4d deposited in 6.81% of the LN patients, which was related to the disease activity (13). Although we found a higher rate of C4d deposition in PTCs, we could not confirm a significant association between PTC-C4d deposition and disease activity.

Importantly, LN patients with TBM-C4d deposition presented with more severe clinico-pathological features such as a higher disease activity, worse renal function, lower complement level, greater risk of acute kidney injury, and higher AI and CI scores. As the TBM immune complex deposition is closely associated with disease activity and LN progression (36), we proposed that TBM-C4d deposition could reflect the *in situ* complement activation of the classical or lectin pathway, thus aggravating local inflammation.

RVLs are of great importance in LN and have prognostic value (24). Previous studies described arteriolar C4d deposition in TMA cases associated with SLE (37, 38). In a recent study done by Mejia-Vilet, although they did not find significant difference in C4d deposition in any compartment between patients with highly active LN and those with concomitant TMA, the latter group prone to have more C4d deposition in all the renal compartments (C4d positivity in different renal compartments in active LN *vs.* active TMA+active LN: glomerular capillaries: 12 *vs.* 14 patients; peritubular capillaries: 8 *vs.* 11 patients; tubular basement membrane: 6 *vs.* 12 patients; arterioles: 4 *vs.* 8 patients). The diverse conclusions between different papers from various cohorts might be attributed to the different sample size and the method of C4d evaluation (39). However, data concerning other RVL types in LN are lacking. We found a close association between arteriolar C4d deposition and different RVLs, including TMA changes. We speculate that some common pathway might be involved in RVL pathogenesis, and the higher rate of arteriolar C4d deposition in patients with RVL suggests that the complement system might be causally involved in its pathogenesis. Interestingly, nearly all the arteriolar C4d positive patients in our study had ICD lesions, so we further stained samples for C1q. As anticipated, 75.5% of the arteriolar C4d- positive patients had C1q deposition, which was in line with previous studies regarding TMA lesions, in which classical pathway activation played a major role (15, 37). Thus, complement activation through the classical pathway was suggested to be a common pathomechanism involved in various types of RVLs. Interestingly, higher rates of IgM and IgA deposition were also found in C4d-positive patients. Chua et al. proposed C4d might also be a consequence of damage rather than an underlying cause. Chronic endothelial cell injury may result in the formation of a duplicate glomerular basement membrane, which could entrap aspecific immunoglobulins and C3, thus mimicking immune complex deposition (37). Interestingly, nine patients without RVLs in our cohort also had arteriolar C4d deposition. We propose that C4d deposition in those patients might result from the non-specific immune complex activation in LN.

Moreover, arteriolar C4d positive patients had a higher rate of combined C3c deposition compared to those who were negative. Both arteriolar C4d- and C3c-positive patients had the worst renal outcomes. Recent work showed that terminal complement complex (C5b-9) deposition in glomeruli did not differ between active and chronic disease in LN (40). In contrast, C3c staining was associated with active disease in LN, which would be a better indicator to identify patients most likely to benefit from complement inhibiting treatment (40). Thus, a local cascade of complement activation cascade might be more likely in patients with arteriolar C4d deposition, inducing RVL progression and indicating ongoing disease activity. So, it might serve as an alternative test to find patients who might benefit from anti-complement therapy.

## Conclusion

In summary, complement factor C4d deposition was common, but its prevalence varied among different renal compartments in patients with LN. TBM-C4d deposition was associated with more severe clinico-pathological features, and arteriolar C4d deposition was closely related to RVLs renal outcomes. Further studies are needed to clarify its actual role in the pathomechanism of LN.

## Data Availability Statement

The original contributions presented in the study are included in the article/**Supplementary Material**. Further inquiries can be directed to the corresponding author.

## Ethics Statement

The studies involving human participants were reviewed and approved by the Research and Ethics Board of the Peking University First Hospital [Approval reference: No. 2017 (1333)]. The patients/participants provided their written informed consent to participate in this study.

## Author Contributions

YD, LW, and YT planned the project, selected patients, interpreted results, and wrote the paper. XY performed analysis of the renal pathology. ZQ and FY planned the project and revised the paper. All authors contributed to the article and approved the submitted version.

## Funding

This work was supported by Grants of National Natural Science Foundation of China (No. 81870479) and Pekin University International Hospital Research Grant (No. YN2020QN01, No. YN2020ZD03).

## Conflict of Interest

The authors declare that the research was conducted in the absence of any commercial or financial relationships that could be construed as a potential conflict of interest.

## Supplementary Material

Supplementary Figure 1 Flow chart of enrollment of lupus nephritis patients and the design of the study. Abbreviations: LN, lupus nephritis.

Supplementary Table 1 Analysis of co-deposition between arteriolar C4d and C1q, C3c and different immunoglobulins (IgG, IgM and IgA) in C4d positive patients.
