# Supplementary figures and images for: The Spectrum of C4d Deposition in Renal Biopsies of Lupus Nephritis Patients
Source: Front Immunol. 2021 Jul 1;12:654652. doi: 10.3389/fimmu.2021.654652 (PMC8281350; doi:10.3389/fimmu.2021.654652)

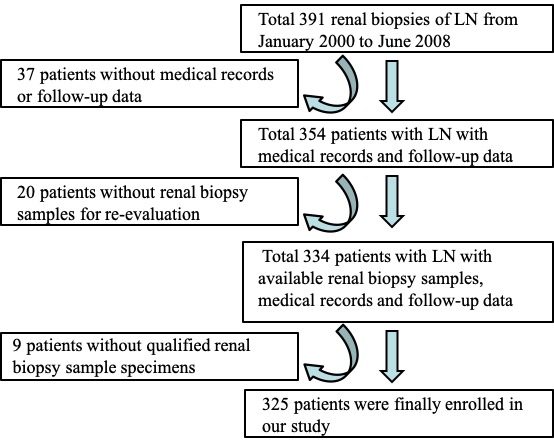

Supplement: Supplementary Figure 1 — Flow chart of enrollment of lupus nephritis patients and the design of the study. Abbreviations: LN, lupus nephritis. [file Image_1.jpeg]
