# Supplementary material for: The Spectrum of C4d Deposition in Renal Biopsies of Lupus Nephritis Patients
Source: Front Immunol. 2021 Jul 1;12:654652. doi: 10.3389/fimmu.2021.654652 (PMC8281350; doi:10.3389/fimmu.2021.654652)
Supplement: Supplementary Table 1 — Analysis of co-deposition between arteriolar C4d and C1q, C3c and different immunoglobulins (IgG, IgM and IgA) in C4d positive patients. [file Table_1.docx]

**Supplementary Material**

**Supplementary Table 1(S1). Analysis of co-deposition between arteriolar C4d and C1q, C3c and different immunoglobulins (IgG, IgM and IgA) in C4d positive patients.**

|  | C3c－ | C3c＋ | *P* | C1q－ | C1q＋ | *P* | IgG- | | IgG+ | *P* | IgM- | IgM+ | *P* | IgA－ | IgA＋ | *P* |
| --- | --- | --- | --- | --- | --- | --- | --- | --- | --- | --- | --- | --- | --- | --- | --- | --- |
| Number of patients | 62 | 40 |  | 25 | 77 |  | 61 | | 41 |  | 64 | 38 |  | 84 | 18 |  |
| AI score  (median, IQR) | 8(2.75-12) | 9(6.25-12) | 0.151 | 8(1.5-11.5) | 9(5.5-12) | 0.255 | 8(3-12) | 9(6.5-12) | | 0.208 | 8(3-11.75) | 10.5(6-12.25) | 0.096 | 8(3.25-12) | 10.5(7-12.25) | 0.053 |
| Endocapillary hypercellularity  (median, IQR) | 2.5(1-3) | 3(2-3) | 0.050 | 2(0.5-3) | 3(2-3) | 0.071 | 3(1-3) | | 3(2-3) | 0.297 | 2(1-3) | 3(2-3) | 0.016 | 2.5(1-3) | 3(3-3) | 0.003 |
| Cellular crescents  (median, IQR) | 2(0-2) | 2(0-4) | 0.118 | 0(0-2) | 2(0-2) | 0.493 | 2(0-2) | | 2(0-4) | 0.096 | 2(0-2) | 2(0-4) | 0.171 | 2(0-2) | 2(0-6) | 0.119 |
| Karyorrhexis/fibrinoid necrosis (median, IQR) | 0(0-2) | 1.5(0-2) | 0.565 | 2(0-2) | 0(0-2) | 0.734 | 1(0-2) | | 0(0-2) | 0.966 | 0(0-2) | 1(0-2) | 0.869 | 0.5(0-2) | 1(0-2) | 0.929 |
| Subendothelial hyaline deposits (median, IQR) | 1(0-2) | 1(0-1.75) | 0.803 | 0(0-1.5) | 1(0-2) | 0.051 | 1(0-2) | | 1(0-1) | 0.807 | 1(0-2) | 1(0-2) | 0.224 | 1(0-2) | 1(1-2.25) | 0.059 |
| S1 (Continued) | | | | | | | | | | | | | | | | |
| Interstitial inflammatory cell infiltration  (median, IQR) | 1(1-2) | 1(1-2) | 0.358 | 1(1-2) | 1(1-2) | 0.248 | 1(1-2) | | 1(1-2) | 0.370 | 1(1-2) | 1(1-2) | 0.052 | 1(1-2) | 1(1-2.25) | 0.118 |
| Glomerular leukocyte infiltration (median, IQR) | 1(0-1) | 1(1-1) | 0.046 | 1(0-1) | 1(0-1) | 0.788 | 1(0-1) | | 1(1-1) | 0.135 | 1(0-1) | 1(0-1) | 0.811 | 1(0-1) | 1(1-1) | 0.173 |
| CI score (median, IQR) | 2.5(2-4) | 3(2-5) | 0.024 | 2(2-3) | 3(2-4.5) | 0.014 | 3(2-4) | | 3(2-5) | 0.140 | 2.5(2-4) | 3(2-5) | 0.052 | 3(2-4) | 3.5(2-5) | 0.217 |
| Glomerular sclerosis (median, IQR) | 0(0-1) | 1(0-1) | 0.396 | 0(0-1) | 1(0-1) | 0.191 | 0(0-1) | | 0(0-1) | 0.793 | 0(0-1) | 1(0-1) | 0.112 | 0(0-1) | 0(0-1) | 0.845 |
| Fibrous crescents  (median, IQR) | 0(0-0) | 0(0-0) | 0.547 | 0(0-0) | 0(0-0) | 0.020 | 0(0-0) | | 0(0-0) | 0.280 | 0(0-0) | 0(0-0) | 0.820 | 0(0-0) | 0(0-0) | 0.263 |
| Tubular atrophy  (median, IQR) | 1(1-2) | 1(1-2) | 0.049 | 1(1-1) | 1(1-2) | 0.045 | 1(1-1.5) | | 1(1-2) | 0.060 | 1(1-1.75) | 1(1-2) | 0.023 | 1(1-2) | 1(1-2) | 0.099 |
| Interstitial fibrosis (median, IQR) | 1(1-1) | 1(1-2) | 0.027 | 1(1-1) | 1(1-1.5) | 0.093 | 1(1-1) | | 1(1-2) | 0.021 | 1(1-1) | 1(1-2) | 0.072 | 1(1-1) | 1(1-2) | 0.031 |

Note: AI, activity indices; CI, chronicity indices; s.d., standard deviation; IQR, interquartile range.
